# Cone degeneration is triggered by the absence of USH1 proteins but prevented by antioxidant treatments

**DOI:** 10.1038/s41598-018-20171-0

**Published:** 2018-01-31

**Authors:** Alix Trouillet, Elisabeth Dubus, Julie Dégardin, Amrit Estivalet, Ivana Ivkovic, David Godefroy, Diego García-Ayuso, Manuel Simonutti, Iman Sahly, José A. Sahel, Aziz El-Amraoui, Christine Petit, Serge Picaud

**Affiliations:** 1 Sorbonne Université, INSERM, CNRS, Institut de la Vision, 17 rue Moreau, F-75012 Paris, France; 2 Departamento de Oftalmología, Facultad de Medicina, Universidad de Murcia, and Instituto Murciano de Investigación Biosanitaria- Hospital Virgen de la Arrixaca (IMIB-Arrixaca), 30100 Murcia, Spain; 3 Génétique et Physiologie de l’Audition, Institut Pasteur, 75015 Paris, France; 4 UMRS 1120, Institut National de la Santé et de la Recherche Médicale, 75015 Paris, France; 5 CHNO des Quinze-Vingts, DHU Sight Restore, INSERM-DGOS CIC 1423, 28 rue de Charenton, F-75012 Paris, France; 6 Fondation Ophtalmologique Adolphe de Rothschild, 75019 Paris, France; 7 Académie des Sciences, 75006 Paris, France; 8 Collège de France, 75005 Paris, France

## Abstract

Usher syndrome type 1 (USH1) is a major cause of inherited deafness and blindness in humans. The eye disorder is often referred to as retinitis pigmentosa, which is characterized by a secondary cone degeneration following the rod loss. The development of treatments to prevent retinal degeneration has been hampered by the lack of clear evidence for retinal degeneration in mutant mice deficient for the Ush1 genes, which instead faithfully mimic the hearing deficit. We show that, under normal housing conditions, *Ush1g*^−/−^ and *Ush1c*^−/−^ albino mice have dysfunctional cone photoreceptors whereas pigmented knockout animals have normal photoreceptors. The key involvement of oxidative stress in photoreceptor apoptosis and the ensued retinal gliosis were further confirmed by their prevention when the mutant mice are reared under darkness and/or supplemented with antioxidants. The primary degeneration of cone photoreceptors contrasts with the typical forms of retinitis pigmentosa. Altogether, we propose that oxidative stress probably accounts for the high clinical heterogeneity among USH1 siblings, which also unveils potential targets for blindness prevention.

## Introduction

Usher syndrome type 1 (USH1) is the most severe form of inherited deafness and blindness in humans^1–3^ with a prevalence recently estimated from 1/6000 to 1/10000. It leads to congenital profound hearing loss, severe balance problems, and prepubertal visual deficits ultimately leading to retinal degeneration. Although the dysfunctional sensory abilities of patients critically impede, both physically and socially, the patients in their daily life, the underpinnings of the Usher syndrome remain poorly understood. Though the associated congenital deafness is not preventable, the visual defects are delayed and therefore offer the possibility for a therapeutic window. Clinical heterogeneity in retinal degeneration has been observed among USH1 siblings, suggesting a possible modulation of the phenotype by genetic background and/or environmental factors^4–9^. The six identified causal genes for USH1 code for proteins expressed in hair cells and at the connecting cilium in both rod and cone photoreceptor cells; these are: myosin VIIa (USH1B), harmonin (USH1C), cadherin-23 (USH1D), protocadherin-15 (USH1F), and sans (USH1G) (see http://hereditaryhearingloss.org/). The USH1 proteins belong to a subcellular network and play an essential role in hair cell mechano-electrical transduction^1–3^. In contrast, their role in photoreceptors is poorly understood due to the lack of an appropriate animal model presenting with visual defects (see below). The eye disorder is often referred to as retinitis pigmentosa, which is classically thought to lead to blindness via an early loss of rod photoreceptors and a secondary cone degeneration (https://www.nidcd.nih.gov/health/usher-syndrome)^10,11^. This secondary cone degeneration was recently attributed to either the loss of rod-derived cone viability factor (RdCVF)^12^, or to the increase exposure to oxygen of cone photoreceptors in the thinning outer nuclear layer^13^. However, Usher syndrome differs from most other forms of retinitis pigmentosa because expression of the mutated USH1 gene is not limited to rods but is also present in cones^1^. Clarifying the mechanisms underlying cone degeneration in USH syndrome is essential for the development of therapeutic treatments, especially as successful preservation of more than 10% of cones at the fovea could be sufficient to preserve a normal visual acuity in patients^14^.

Mutant mice deficient for any one of the Ush1 genes are profoundly deaf, but none display clear evidence of spontaneous photoreceptor degeneration^15–25^. The absence of a retinal phenotype appears to be independent of the mouse background (albino or pigmented)^15–25^, and was recently attributed to the reduced number of calyceal processes^26^, microvillar structures surrounding the base of the outer segment in primate photoreceptors^27^. Nevertheless, few Ush1 mutant mice still displayed some retinal abnormalities. Under normal housing conditions, rod degeneration was only reported in knock-in mice expressing the Acadian USH1C gene^28^. The myosinVIIa-defective mice, shaker-1 mice, show altered opsin transport through the photoreceptor cilium to the outer segment^18^, aberrant melanosome localization and motility in the retinal pigment epithelium^29,30^, defective phagosome localization and digestion^31^, and RPE65 dysfunction and delocalization in the retinal pigment epithelium^32^. Electroretinograms were often reduced in amplitude in many mouse models suggesting minor photoreceptor dysfunction^16,19,21,30^. Studies have also housed mice in different light conditions to determine if photoreceptors were more susceptible to high photooxidative stress in Ush1 mouse models. These studies produced conflicting findings with myosinVIIa-defective shaker1 mice being either more resistant to acute light damage than controls^32^, or showing rod degeneration upon exposure to moderate light (1500 lux) for 6 months or to constant light for 6 days^33^.

To study these issues, we developed two murine models of USH1 mice, defective for either Ush1c or Ush1g genes^34,35^, which encode the two scaffolding proteins, harmonin and Sans, respectively. We investigated the fate of their photoreceptors under varying housing and diet conditions, on a different background. In these two new models, we found greater changes occurring in cone photoreceptors under a set of specific conditions.

## Results

### No retinal phenotype in the C57Bl/6J background

**Figure 1:**
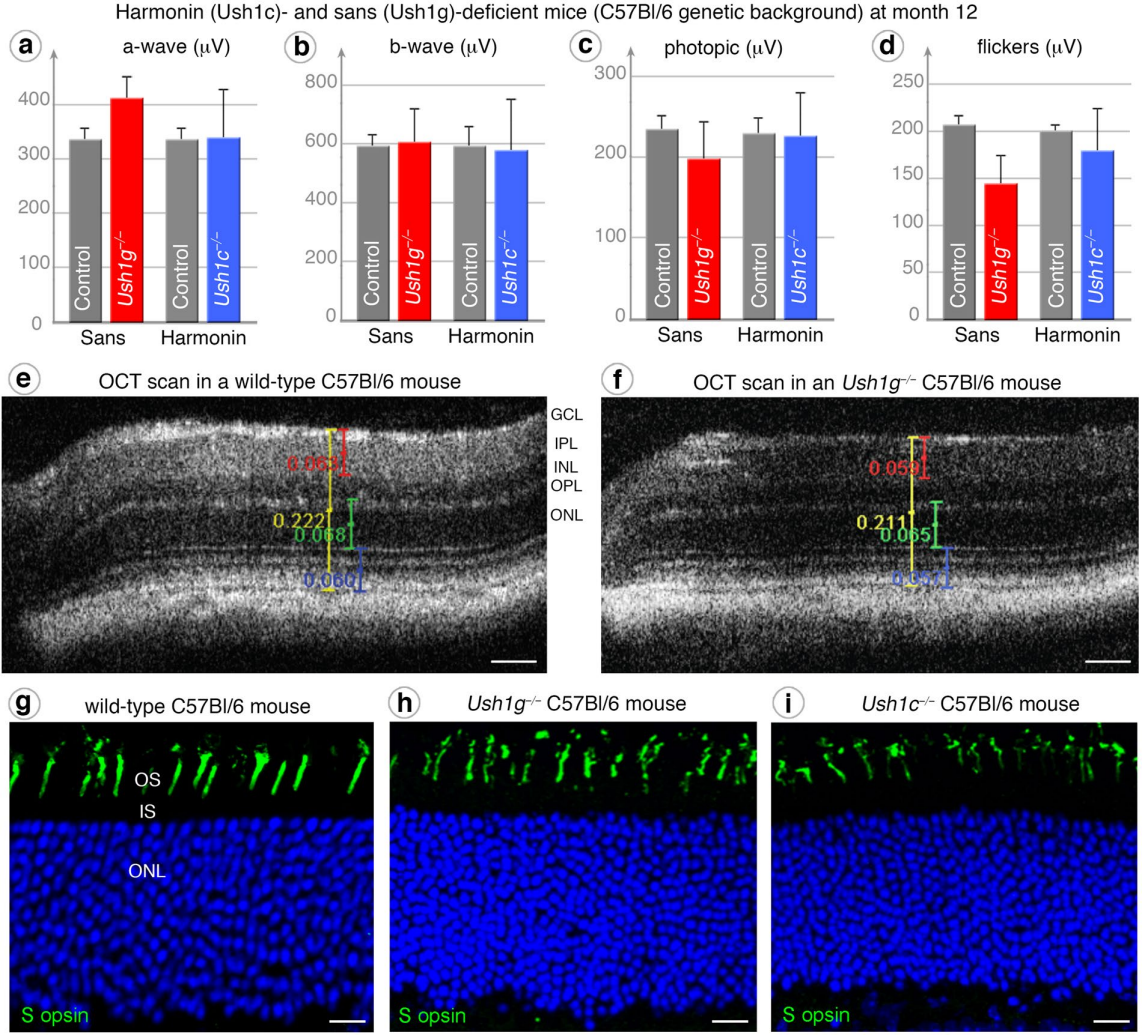
Absence of a retinal phenotype in pigmented (C57Bl/6J genetic background) Ush1c−/− and Ush1g−/− mice. (**a**–**d**) Electroretinogram (ERG) measurements performed at month 12 on wild-type control (grey), *Ush1g*^−/−^ (red) and *Ush1c*^−/−^ (blue) C57Bl/6 J mice showing no significant difference in the amplitudes of ERG responses, regardless of the test conditions: dark-adapted (**a** and **b**), photopic (**c**) or flickers (**d**). (**e**,**f**) OCT scans reveal no difference in retinal layers thickness of C57Bl/6 J *Ush1g*^−/−^ mice (**f**) compared to control mice (**e**). In the retina of *Ush1g*^−/−^ and *Ush1c*^−/−^ C57Bl/6 J mice, the immunolabeling for cone blue opsin is well restricted to unaffected outer segments (**h**,**i**), resembling those observed in control mice (**g**). The scale bars represent 50 µm in (**e** and **f**), and 10 µm in (**g**–**i**).

We first analyzed the retinal phenotypes of the two Ush1 null mouse models generated, both in the C57BI/6 J strain, one of which lacked sans (*Ush1g*^−/−^)^35^, whereas the other lacked all harmonin isoforms (*Ush1c*^−/−^)^34^. Functional ERG measurements at twelve months of age (12 mo), under dark -adapted (Fig. 1a,b), photopic (Fig. 1c) or flicker (Fig. 1d) conditions, indicated that rod and cone functions were normal in the two mutants. I*n vivo* optical coherence tomography (OCT) (Fig. 1e,f) and histological immunostaining (Fig. 1g–i) showed the retina to have a normal thickness and architecture, with a normal photoreceptor organization. This absence of morphological or functional abnormalities in *Ush1g*^−/−^ and *Ush1c*^−/−^ C57Bl/6 J mice was consistent with the lack of retinal degeneration observed in previously reported harmonin mouse models^15–17^.

### Photoreceptor dysfunction in albino animals

**Figure 2:**
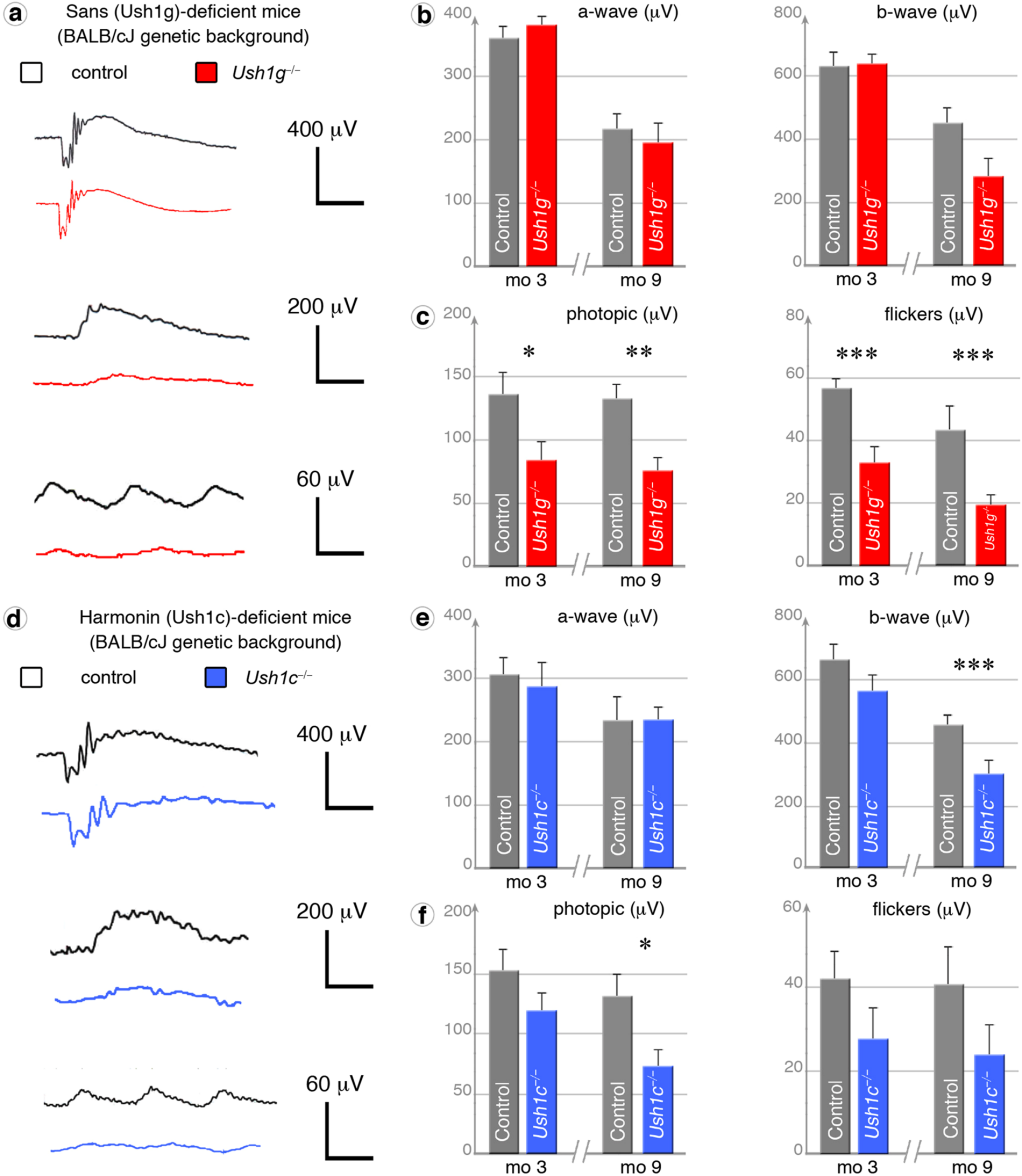
Cone dysfunction in albino Ush1c^−/−^ and Ush1g^−/−^ BALB/cJ mice. (**a**) ERG recordings in three-month-old (mo 3) and nine-month-old (mo 9) Ush1g^−/−^ (red) and control (grey) BALB/cJ mice under dark-adapted (0.2 cds/m2), photopic and flicker (10 Hz) conditions. (**b**,**c**) Quantification of dark-adapted a-wave (rod hyperpolarization) and b-wave (bipolar neuron depolarization) ERG amplitudes (**b**), photopic ERG amplitudes and flicker amplitudes (**c**, cone pathway) at mo 3 and mo 9, showing significantly lower amplitudes in the mutant mice on photopic ERGs (mo 3: *p* = 0.025, *n* = 8; mo 9: *p* = 0.0011, *n* = 10), flickers (mo 3: *p* = 0.0012; mo 9: *p* = 0.0023) and dark-adapted ERG b-wave amplitudes (mo 9: *p* < 0.05). (**d**) ERG recordings in *Ush1c*^−/−^ (blue) and control (grey) BALB/cJ mice at mo 9 under dark-adapted, photopic and flicker conditions. (**e**,**f**) Quantification of a-wave and b-wave amplitudes on dark-adapted (0.2 cds/m2) ERGs (**e**), amplitudes on photopic ERG and flicker ERGs (10 Hz) (**f**) at mo 3 and mo 9, showing significantly lower amplitudes of dark-adapted b-wave (*p* = 0.0036, *n* = 8) and photopic ERG (*p* = 0.0011, *n* = 7) responses in mutant mice at mo 9. The data shown are means ± SEM. (*), (**), and (***) denote *p* < 0.05, *p* < 0.01, and *p* < 0.005, respectively (Student’s *t*-test).

Unlike patients, who experience variations in light exposure during their daily life, mice used as experimental models are reared in animal facilities in which light intensities are fixed at very low levels (below 100 lux in cages). We investigated the possible effects of light exposure on photoreceptor degeneration by backcrossing the *Ush1g*^−/−^ and *Ush1c*^−/−^ C57BL/6J mice onto an albino BALB/cJ background, thereby increasing the range of irradiance levels reaching the retina in these mice. Indeed, the absence of pigment in the retina of albino mice has been shown to result in levels of retinal irradiance 70 times higher than those in pigmented mice^36^. Despite no detectable morphological modification by *in vivo* OCT examination (Fig. S1), functional assessment (Figs 2 and S2) of the cone pathway showed the photopic ERG amplitudes in *Ush1g*^−/−^ BALB/cJ mice became 38% (p < 0.05 at three months) and 43% (*p* < 0.005 at nine months) lower as compared to age-matched control BALB/cJ mice (Fig. 2a and c). Cone pathway dysfunction was confirmed by 10 Hz flicker-ERG recordings (Fig. 2c) performed at three (32.5 ± 5.6 μV; *p* < 0.005) and nine (18.9 ± 3.4 μV; *p* < 0.005) months (comparisons with control BALB/cJ mice: 56.6 ± 3.5 μV at three months and 43.2 ± 7.9 at nine months) and by 20 Hz flicker-ERG recording (Fig. 2b). We also investigated the rod pathway under dark-adapted conditions, and revealed smaller amplitudes of ERG b-wave (0.2 cds/m2) at nine months in *Ush1g*^−/−^ BALB/cJ mice (Fig. 2a,b, *p* < 0.05). Difference was not statistically different at 0.1 cds/m2 (Fig. 2a). Similar experiments in *Ush1c*^−/−^ BALB/cJ mice at nine months of age showed that the amplitudes of photopic ERGs and dark-adapted b-waves were 44% (*p* < 0.05) and 33% (*p* < 0.05) lower, compared to control mice (Fig. 2d–f). Cone pathway dysfunction was confirmed by 20 Hz flickers-ERG recordings (Fig. S2d). Change to the albino genetic background in the sans or harmonin mouse models leads to photoreceptor dysfunction, which appears first in cones.

### Cone morphological changes

**Figure 3:**
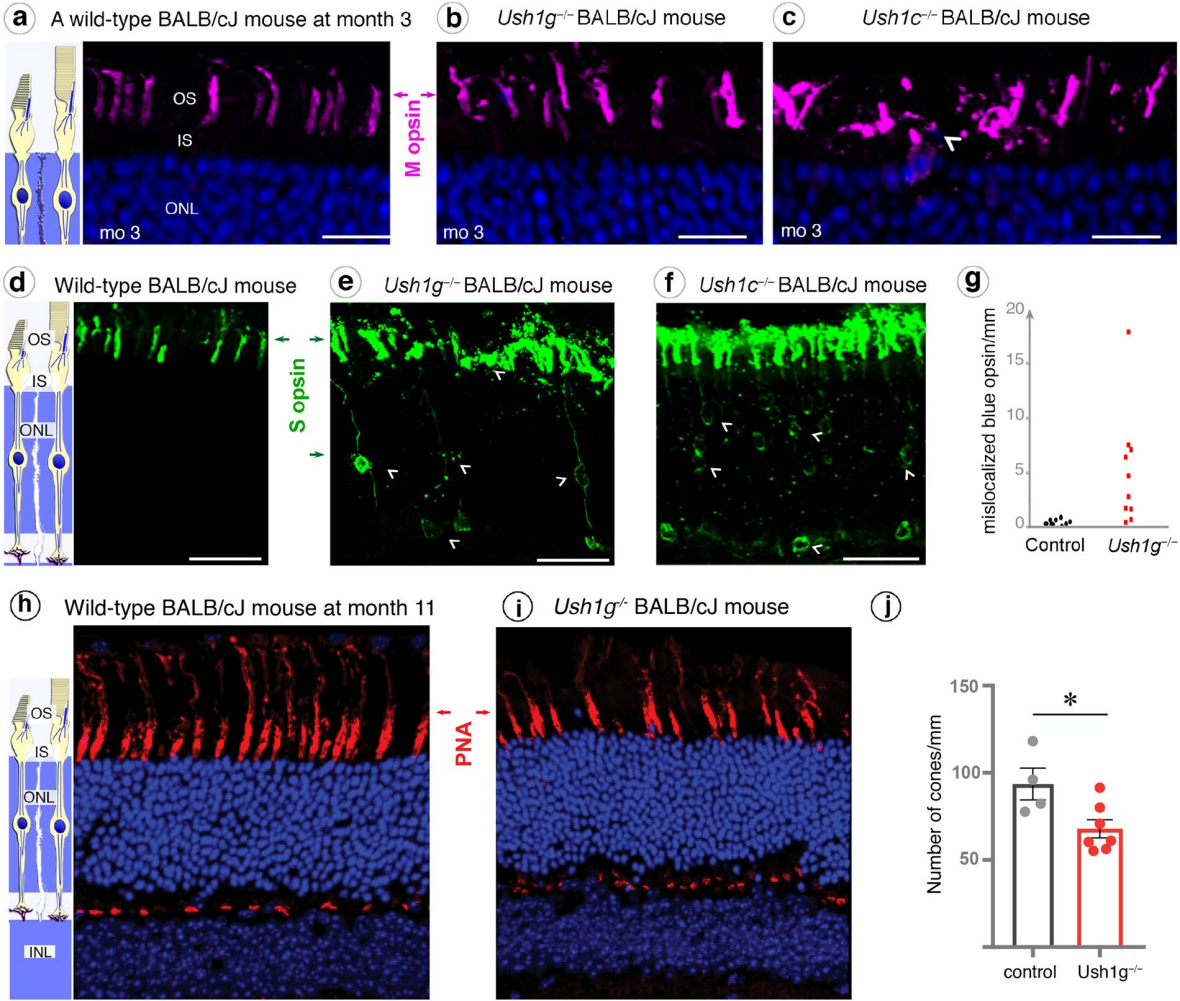
Mislocalization of cone opsins in *Ush1c*^−/−^ and *Ush1g*^−/−^ BALB/cJ mice and cone loss in *Ush1g*^−/−^ BALB/cJ mice. **(a**–**c**) Immunolabeling of M opsin on retinal cross-sections at month 3 (mo 3), showing that the outer segments (OS) are normal in shape in wild-type (**a**) and *Ush1g*^−/−^ BALB/cJ mice (**b**), whereas the outer segments in *Ush1c*^−/−^ BALB/cJ mice are disorganized (**c**, white arrowhead). (**d**–**g**) The S opsin immunolabeling, which was restricted to the outer segment in wild-type BALB/cJ mice (**d**) revealed alterations to the outer segments in the two mutant mice, with mislocalized labeling extending over the entire photoreceptor cell body (white arrowheads, **e** and **f**). (**g**) Quantification of cone cells with mislocalized immunolabeling for S opsin (number of cells/mm retinal cross-section, p < 0.0001, Student’s *t*-test, n = 10 in each group). (**h**–**j**) Lectin PNA staining on retinal cross-sections in 11-month-old control (**h**) and *Ush1g*^−/−^ (**i**) BALB/cJ mice showing a reduction in the number of PNA-stained cone photoreceptor inner/outer segments in *Ush1g*^−/−^ (red) BALB/cJ mice (**j**, number of cells/mm retinal cross-section, p < 0.05 n = 4 control animals, n = 7 *Ush1g*^−/−^ mice). The scale bars represent 5 µm in (**a**–**c**), 10 µm in (**d**–**f**) and 50 µm in (**h** and **i**). ONL: outer nuclear layer, INL: inner nuclear layer, IPL: inner plexiform layer.

We then investigated the subcellular distribution of photoreceptor-specific markers in 3 months old *Ush1g*^−/−^ BALB/cJ mice. The distribution of medium wavelength sensitive opsin (M opsin) showed normal localization in *Ush1g*^−/−^ BALB/cJ mice and normally shaped outer segments of the photoreceptor cells (Fig. 3a,b). By contrast, immunolabeling for the short wavelength sensitive opsin 1 (S opsin) was spread over the entire cell body and synaptic terminals (Fig. 3e, white arrowheads), rather than being restricted to the outer segment in blue cones (Fig. 3d). The quantification of blue cones with a mislocalized S-opsin indicated a great difference between *Ush1g*^−/−^ BALB/cJ mice and control animals (Fig. 3g, n = 10 in both groups, p < 0.0001). In addition, the S opsin labeled outer segments were shorter and thicker in *Ush1g*^−/−^ BALB/cJ mice compared to age-matched control mice (Fig. 3d,e). A similar mislocalization of S opsin and shorter outer segments were observed in *Ush1c*^−/−^ BALB/cJ mice at nine months (Fig. 3f, white arrowheads). In addition, the outer segment was also thicker in the M-cones of these mutants compared to that in control mice (Fig. 3c). These results suggest that the absence of *Ush1* genes triggers S opsin delocalization and changes in S-cones outer segment morphology.

### Photoreceptor apoptosis and retinal gliosis

**Figure 4:**
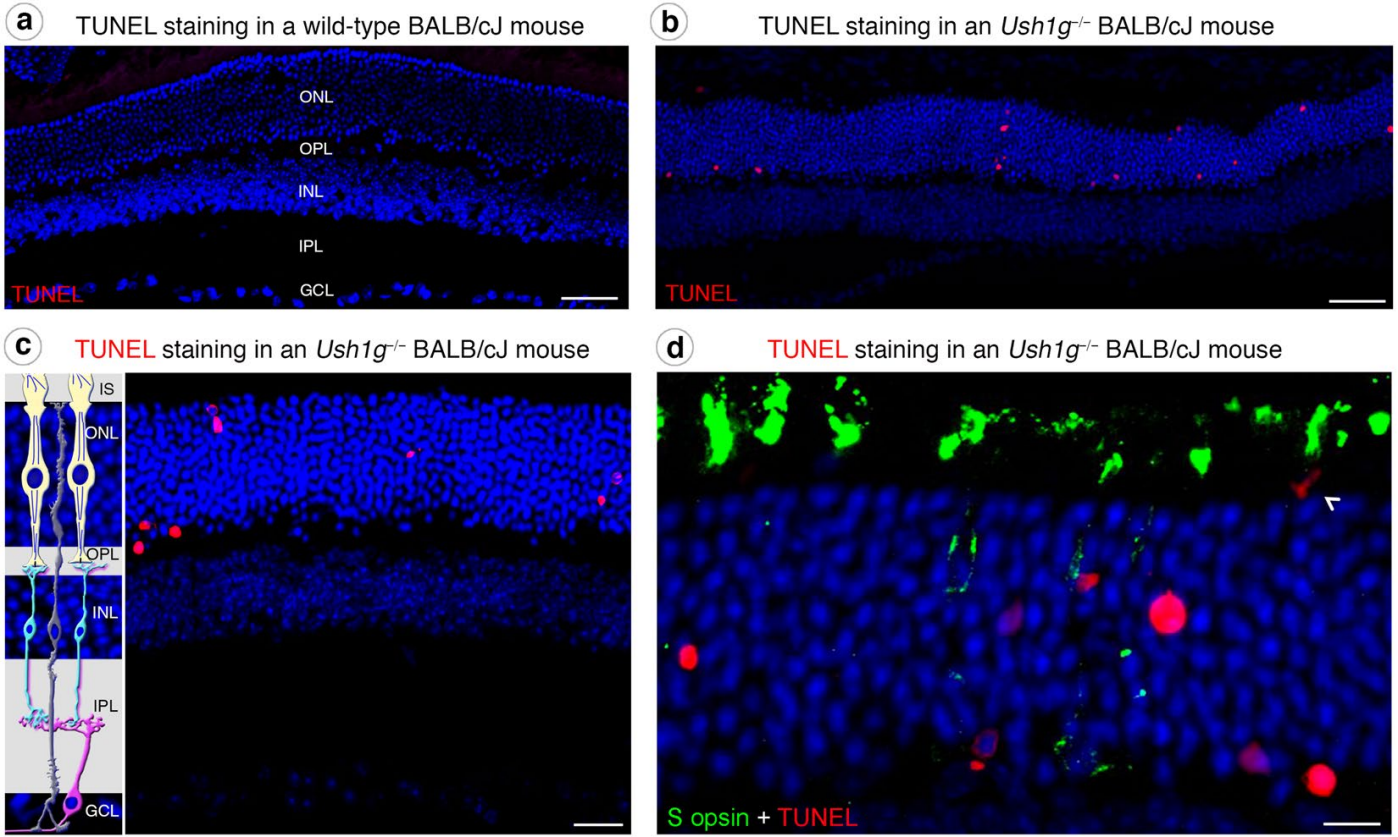
Photoreceptor apoptosis in *Ush1g*^−/−^ BALB/cJ mice. (**a**–**d**) TUNEL staining in wild-type (**a**) and *Ush1g*^−/−^ (**b**–**d**) BALB/cJ mice at 3 mo. Apoptotic cells (labeled in red) are detected in the outer nuclear layer (ONL) of *Ush1g*^−/−^ BALB/cJ retina (**b**–**d**), whereas such cells were completely absent from the retina of control BALB/cJ mice (**a**). Note the apposition of the TUNEL labeling and the mislocalized S opsin immunolabeling (white arrowhead, **d**). The scale bars represent 50 µm in (**a** and **b**), 20 µm in (**c**), and 15 µm (**d**). OS: outer segment, INL: inner nuclear layer, IPL: inner plexiform layer.

**Figure 5:**
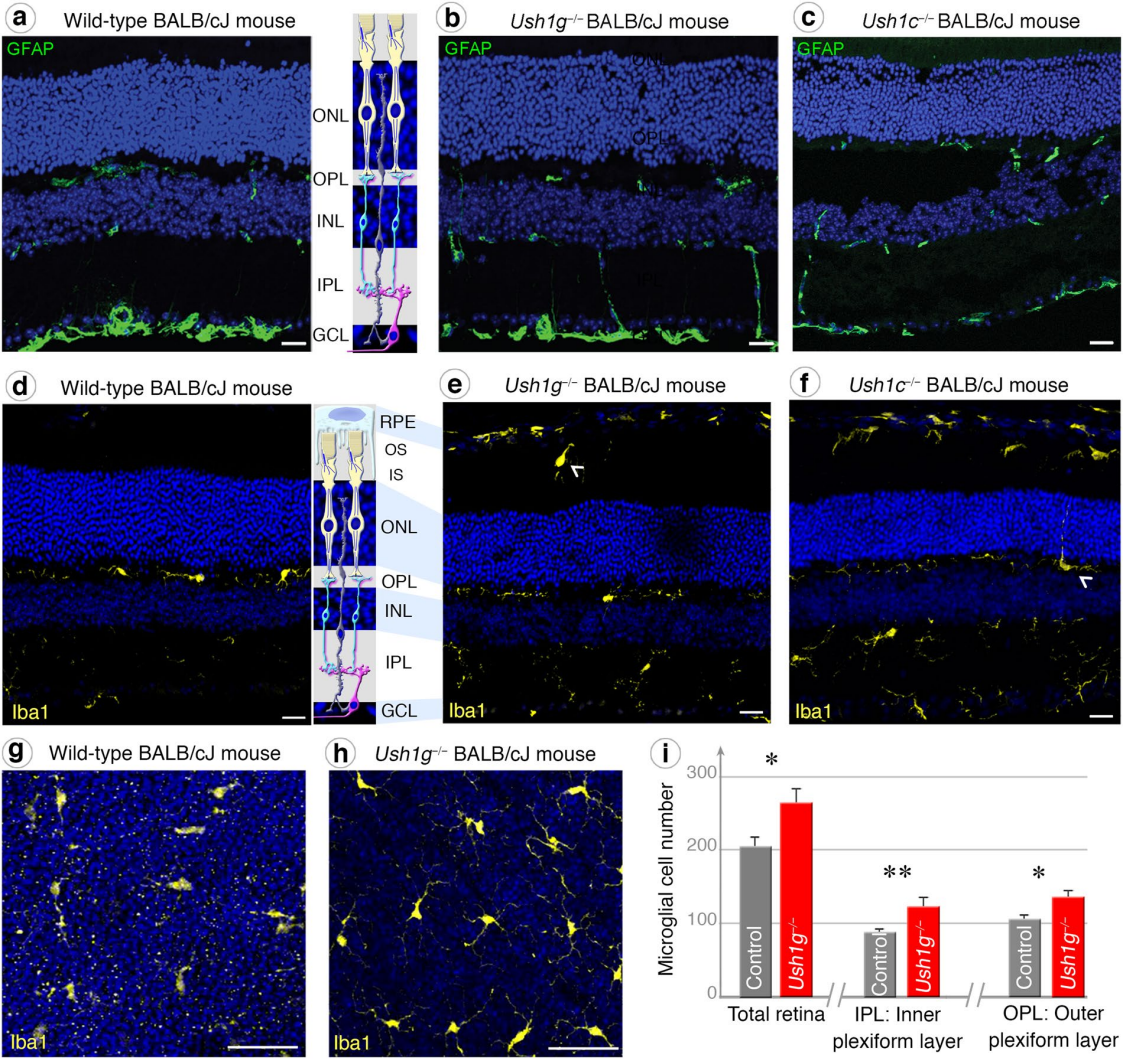
GFAP and Retinal gliosis in Ush1c^−/−^ and Ush1g^−/−^ BALB/cJ mice. (**a**–**c**) GFAP immunolabeling on retinal cross-sections display similar distribution patterns between control (**a**), *Ush1g*^−/−^ (**b**) and *Ush1c*^−/−^ (**c**) BALB/cJ mice. (**d**–**h**) Iba1-immunopositive microglial cells on retinal sections. Microglial cells are regularly distributed in the inner (IPL) and outer (OPL) plexiform layers in control animals (**d**). In *Ush1g*^−/−^ (**e**) and *Ush1c*^−/−^ (**f**) BALB/cJ mice, activated microglial cells are also present in the regions of outer (OS) and inner (IS) segments of photoreceptor cells (white arrowhead, **e**). In (**f**), a microglial cell with a process extending in the outer nuclear layer (ONL) (white arrowhead). (**g** and **h**) Flat-mounted retinas of wild-type (**g**) and *Ush1g*^−/−^ (**h**) BALB/cJ mice. The bulging and multiplication of cell bodies shown in the IPL and OPL of *Ush1g*^−/−^ mice (**h**) is a feature of activated microglial cells. (**i**) Quantification on total flat-mounted retinas, comparing *Ush1g*^−/−^ mice with age-matched controls, showed an increase in the numbers of microglial cells in the IPL, OPL, and throughout the entire retina. The data shown are means ± SEM. *p < 0.05, **p < 0.01 (Student’s *t*-test). The scale bars represent 15 µm in (**a**–**f**) and 50 µm (**g** and **h**). RPE: retinal pigment epithelium cell, INL: inner nuclear layer.

Following these molecular and morpho-functional changes in photoreceptors, we examined the presence of apoptotic cells in the outer nuclear layer of our two Usher mouse models. TUNEL assays on retinal cross-sections revealed positive staining strictly located in the outer nuclear layer of 3-month-old *Ush1g*^−/−^ BALB/cJ mice, demonstrating that apoptosis selectively affected photoreceptors (Fig. 4a–d). Cone quantification using Lectin PNA marker on retinal cross-section showed a significant drop in the number of cone photoreceptors in 11-month-old *Ush1g*^−/−^ BALB/cJ mice (Fig. 3h–j). We characterized this degenerative process further by looking for evidence of reactive gliosis. Although changes in GFAP expression by macroglial Müller cells were difficult to demonstrate as an evidence of reactive gliosis (Fig. 5a–c), the morphological changes of microglial cells and their abnormal migratory behavior provided obvious evidence of the reactive gliosis. The Iba1-immunoreactive microglial cells in the retinas of control BALB/cJ mice had features typical of the resting state, including long thin neurites (Fig. 5d and g). By contrast, the Iba1-immunoreactive cells of *Ush1g*^−/−^ and *Ush1c*^−/−^ BALB/cJ mice became ameboid, invading the outer nuclear layer, the layers of the inner and outer segments of photoreceptors (Fig. 5e,f and h, white arrowheads). Also, the numbers of microglial cells overall the retina or in the inner plexiform layer were 30% and 40% higher, respectively, in *Ush1g*^−/−^ BALB/cJ mice compared to age-matched BALB/c control mice (Fig. 5i, *p* < 0.05). These changes in the shape and number of retinal microglial cells in *Ush1c*^−/−^ and *Ush1g*^−/−^ mice confirmed that photoreceptor degeneration was occurring in these two mouse mutants.

### Housing under darkness prevents the photoreceptor degeneration

**Figure 6:**
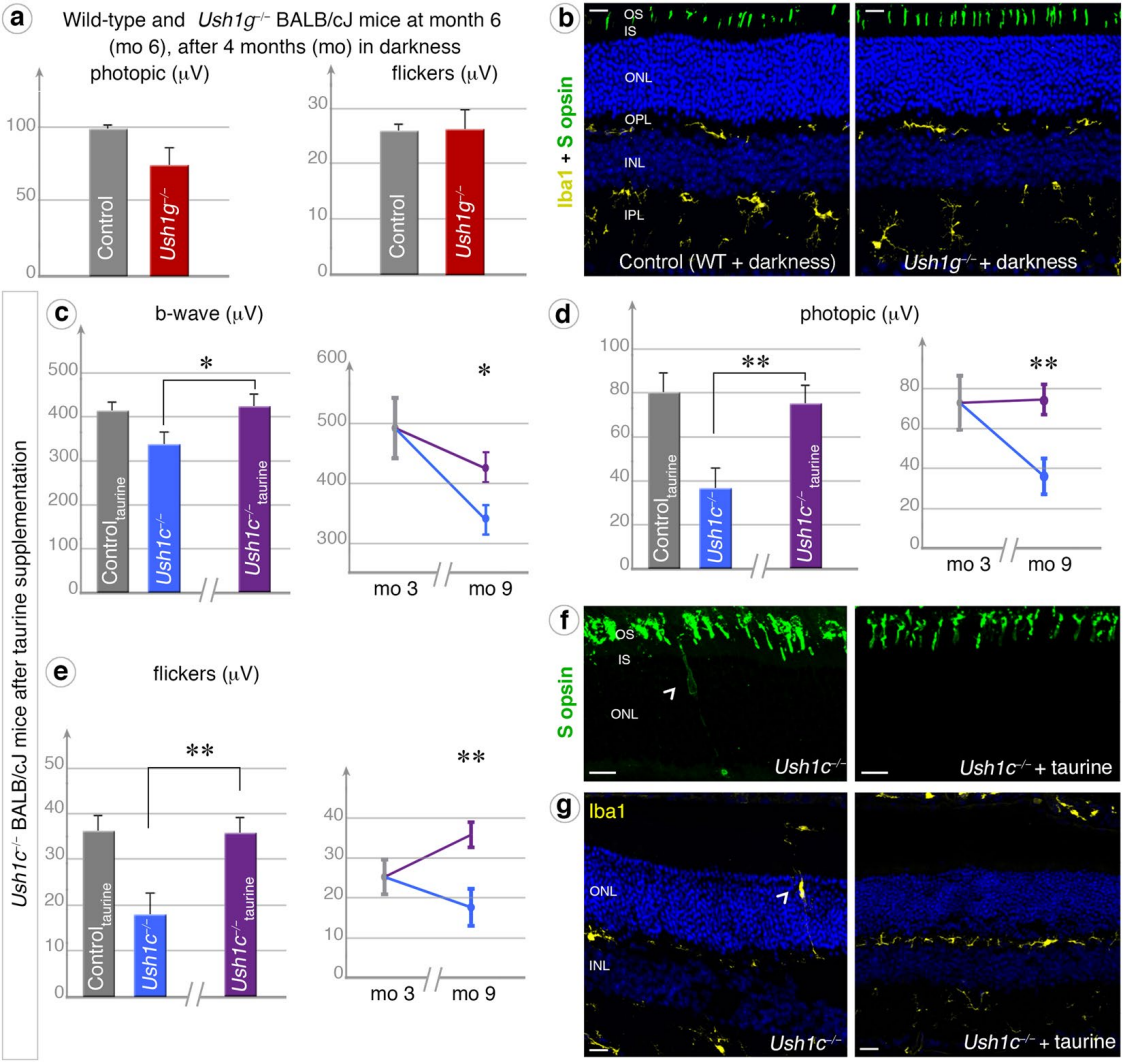
Darkness and taurine supplementation prevent retinal degeneration in Ush1-deficient mice. (**a**,**b**) Being kept in the dark for four months (from mo 2 to mo 6) prevented the occurrence of Ush1 abnormal phenotype in *Ush1g*^−/−^ BALB/cJ mice (dark red graphs), as indicated by the lack of significant difference in photopic and flicker ERG responses, the normal localization of blue opsin in the outer segment (OS), and the even distribution of microglial cells between mutant and control BALB/cJ mice (**b**). (**c**–**g**) The Ush1 retinal phenotype was also rescued by antioxidant (taurine) supplementation. ERG measurements on 9-month-old *Ush1c*^−/−^ BALB/cJ mice treated with 0.1 M taurine (purple graphs) showed that this treatment significantly prevented the loss of retinal function. In all dark-adapted (**c**), photopic (**d**) and flicker (**e**) conditions, the results obtained were similar for treated mutant mice and age-matched BALB/cJ control mice (histograms: *n* = 5 for all conditions). Treatment also prevented a decrease in these parameters during aging, from three (mo 3) to nine months (mo 9), in *Ush1g*^−/−^ mice (curves). Taurine supplementation also prevented the mislocalization of S opsin (**f**) and microglial cells (**g**) observed in *Ush1c*^−/−^ mice not treated with taurine. The data shown are means ± SEM. *p < 0.05, **p < 0.01, ***p < 0.005; (Student’s *t*-test). The scale bars represent 15 µm in (**b**) and (**g**), and 10 µm in (**f**).

We investigated whether the retinal phenotype uncovered in the albino BALB/cJ background resulted from greater exposure to light, by keeping the *Ush1g*^−/−^ albino mice in total darkness between the ages of two and six months, as this phenotype was already evident at three months in mice exposed to light (Fig. 2). In dark-reared animals, ERG measurements showed no dysfunction of rod and cone photoreceptors at three months (Fig. 6a) by contrast to animals reared under standard lighting conditions (Fig. 2). After four months in total darkness, the cone photoreceptors displayed no morphological changes or opsin mislocalization (Fig. 6b). These findings suggested that light exposure promotes the degeneration of photoreceptors in Ush1 mouse mutants, supporting our primary hypothesis.

### Taurine supplementation and special diet prevent the photoreceptor degeneration

The oxidative stress resulting from light exposure may induce photoreceptor degeneration. This prompted us to investigate whether antioxidant treatment could reverse or slowdown the retinal degenerative process. We decided to use the antioxidant taurine as it is required to maintain the survival of photoreceptor cells, especially cones^37,38^ under light exposition^39,40^. We administered taurine to *Ush1c*^−/−^ BALB/cJ mice in drinking water for six months (from month 3 to month 9). After oral taurine administration for six months, ERG amplitudes under dark-adapted, photopic and flicker conditions were nearly normal in *Ush1c*^−/−^ BALB/cJ mice, as shown by comparisons with age-matched BALB/cJ mice (Fig. 6c–e). Taurine treatment prevented both the thickening of cone outer segments, and the mislocalization of cone S opsin (Fig. 6f, right panel). Finally, a few microglial cells were present in the vicinity of the retinal pigment epithelium, but almost all microglial cells were normally targeted to the inner and outer plexiform layers, and, unlike in the *Ush1c*^−/−^ BALB/cJ mice (arrowhead, Fig. 6g left panel), these cells were entirely absent from the photoreceptor outer nuclear layer (Fig. 6g, right panel). Together, these findings suggest that the retinal degeneration observed in *Ush1c*^−/−^ BALB/cJ mice can be prevented by taurine supplementation.

**Figure 7:**
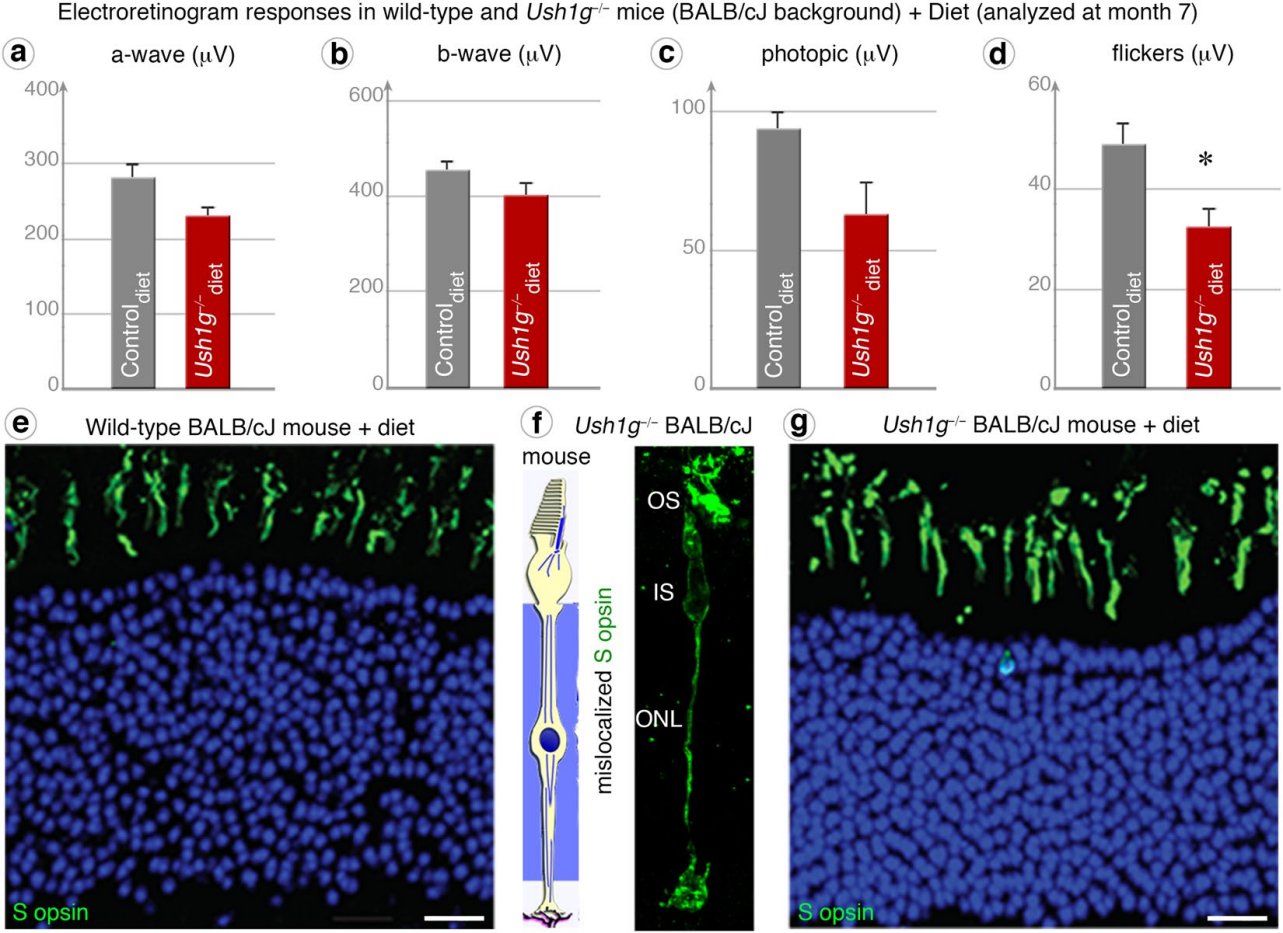
Special diet prevents cone dysfunction and opsin mislocalization. (**a**–**c**) In ERG measurements under dark-adapted (**a** and **b**), photopic (**c**) conditions at month 7, the results obtained for *Ush1g*^−/−^ BALB/cJ mice fed the RM3 diet (a-wave: *p* = 0.1475, b-wave: *p* = 0.2744, photopic: *p* = 0.0806; see Methods) (dark red graphs) were similar to those for age-matched Wild-type (WT) controls (grey). (**d**) However, flicker recordings continued to be significantly lower for *Ush1g*^−/−^ BALB/cJ mice (*n* = 6) than for wild-type control mice (*n* = 8) (*p* = 0.014). (**e**–**g**) Unlike the *Ush1g*^−/−^ BALB/cJ mice (**f**), mutant mice fed by RM3 diet (**g**) display no difference in retinal morphology, as illustrated by immunolabeling for S opsin that is similar to that in age-matched controls (**e**). *p < 0.05 (Student’s *t*-test). The scale bars represent 10 µm in (**e,** and **f**, **g**).

This neuroprotection by taurine suggests that dietary modification could prevent the degenerative process elicited by light in Ush1 mutant mice. *Ush1g*^−/−^ BALB/cJ mice were therefore fed a richer diet (RM3) than the original RM1 diet from postnatal day 15 to month 7 (see methods). ERG measurements at month 7 revealed a near-normal b-wave response in *Ush1g*^−/−^ BALB/cJ mice (dark-adapted conditions: 399.5 ± 27.7 μV; photopic conditions: 62.4 ± 11.8 μV), as shown by comparison with BALB/cJ control mice (dark-adapted conditions: 453.3 ± 38.7 μV; *p* = 0.27; photopic conditions: 93.1 ± 6.9 μV; *p* = 0.08) (Fig. 7a–c). The difference between *Ush1g*^−/−^ BALB/cJ mice and control BALB/cJ mice was significant only for flickers (32.2 ± 4.1 μV in the mutant vs. 48.6 ± 4.4 μV in the control; *p* = 0.014; Fig. 7d). Molecular and histological analyses showed that the S opsin protein localization and the blue cone outer segment morphology were normal (Fig. 7e and g). Although the animal number in each group was rather limited and cannot guaranty the lack of significant difference between the supplemented group and regular diet, these findings suggest that nutritional factors like taurine, can modify the rate of photoreceptor degeneration in Ush1 animal models.

## Discussion

We observed photoreceptor degeneration in two albino Usher mouse models whereas no degeneration was detected in pigmented animals. Morphological and functional changes were greater in cone photoreceptors, suggesting that their degeneration was not dependent upon rod loss as in other models of retinitis pigmentosa. Keeping USH1 mutant albino animals in darkness or supplementing them with the antioxidant taurine preserved the morpho-functional properties of photoreceptors, therefore supporting a potential role of photo-oxidative stress in their degenerative process.

Ever since the identification of the first USH1 gene^41^, the pathological processes underlying visual impairment in USH1 have remained elusive. We recently showed that all the USH1 proteins are colocalized in the calyceal processes — microvillus-like structures emerging from the photoreceptor inner segment and surrounding the outer segments — in humans, non-human primates and other species possessing these structures, such as pig and frog^27,42^. This observation suggested that USH1 proteins might play an essential role in the formation and/or maintenance of the calyceal processes, and that the reduced number of calyceal processes in mouse photoreceptors probably accounts for the lack of a retinal degeneration phenotype in Ush1 mutant mice^26,27,42^. Our findings here obtained in highly controlled experimental and genetic conditions, however, suggested that the photoreceptors of Ush mice lacking either harmonin (*Ush1c*^−/−^) or sans (*Ush1g*^−/−^) are nevertheless highly vulnerable to photo-oxidative stress. Although we cannot exclude genetic modifiers in the albino background strain as reported in an inflammatory disease^43^, our results provide evidence for a contribution of light exposure in the expression of the retinal degeneration phenotype. In addition to light exposure, we showed that diet and nutrients are also likely to affect the level of oxidative stress in photoreceptors and thus their dysfunction, explaining thereby why photoreceptor degeneration was not detected in previous studies on albino Usher mouse models^16,17^.

The USH1 proteins have been shown to form a multiprotein complex in the sensory hair cells and photoreceptors^1–3,44^. Our results suggest that the absence of a protein from this complex can increase the sensitivity of the photoreceptor to oxidative stress. We would expect other Ush1 mice (Ush1b^18–20^, Ush1d^21,22^, Ush1f^23,24,45^, Ush1g^25^) to display retinal degeneration under the same experimental conditions. Conflicting findings with respect to oxidative stress induced by light exposure have been reported in the myosin VIIa-defective shaker-1 mice. Lopes *et al*.^32^ found that Ush1b mice were resistant to acute light damage, whilst Peng *et al*.^33^ observed rod photoreceptor degeneration during constant exposure to intense light for six days or long-term daily exposure to moderate light. In contrast to these results and to typical models of retinitis pigmentosa, we showed that cone photoreceptors in the sans- or harmonin-deficient mice were more sensitive to oxidative stress than rods. This therefore suggests that cone degeneration in USH1 patients probably is not a mere secondary effect due to primary dysfunction of rods as in other forms of retinitis pigmentosa. This conclusion is further supported by different abnormalities in cone ERG recordings from USH1 patients with respect to patients with other forms of retinitis pigmentosa^11^, although some heterogeneity was subsequently reported among USH1 patients^10^. Finally, in animal models of retinitis pigmentosa, the degenerative mechanisms of photoreceptors are very different from what we observe here because, even though this degeneration depends on the light regimen^46^, it occurs both in darkness and in pigmented animals. Similarly, in the knock-in mutant mouse bearing the human gain-of-function *USH1C/c.216 G* > *A* mutation, rod degeneration is observed under normal light intensities in the pigmented mixed C57Bl/6 J and 129S6 genetic background^28^. In this model, photoreceptor dysfunction was detected very early whereas the photoreceptor loss started at 6,5 months to be clearly visible at 1 year. No specific examination was provided with regard to cone morphology or function, preventing a fine comparison of the previous study and ours.

Cone photoreceptors are known to be highly sensitive to oxidative stress^47^. The hypersensitivity of blue cones can be explained by their absorption of shorter wavelengths, which convey more energy and thus elicit greater phototoxicity. Early S-opsin cone modifications resemble the Tritan defects (blue cone deficiency) reported also in some USH1 patients^48^. The lack of genotype-phenotype correlation in USH1-3 patients, including monozygotic twins with USH2^49–52^, is consistent with a potential impact of environmental factors on disease progression. The importance of diet and antioxidant intake in retinitis pigmentosa and Usher syndrome has been highlighted by the finding that disease severity is greater in patients with a lower accumulation of dietary-derived macular pigments^53^. Clinical studies will be required to define the impacts of light and diets on retinal degeneration in USH1 patients.

## Methods

### Animals

Animals were housed with a 12-hour dark/12-hour light cycle, or kept in complete darkness, depending on the experimental design, with food and water available *ad libitum*. Animals were fed the RM1 diet, which has a low protein content (12.91% digestible protein; Special Diet Service (SDS) – 801010; http://www.sdsdiets.com/pdfs/RM1-A-P.pdf), except during the dietary intake experiment, in which animals were fed the RM3 diet containing 19.94% digestible protein (Special Diet Service (SDS) – 801030 http://www.sdsdiets.com/pdfs/RM3-A-P.pdf). The *Ush1g*^−/−^ and *Ush1c*^−/−^ albino mice used in the study were backcrossed with BALB/cJ mice seven and six times, respectively. During taurine supplementation experiments, taurine (Sigma-Aldrich) was dissolved in the drinking water of the treated group, at a concentration of 0.1 M, and administered for four months. Six or more animals were randomly assigned in the different group to be able to perform statistical analysis. All experiments were carried out in accordance with European Community Council Directives (86/609/EEC) and the ARVO (Association for Research in Vision and Ophthalmology) statement for the use of animals in ophthalmic and visual research. The present study was ethically approved by the French « Ministère de l’Education Nationale, de l’Enseignement Supérieur et de la Recherche » under the number 02892.02.

### SD-OCT imaging

Pupils were dilated with tropicamide (Mydriaticum, Théa, France) and phenylephrine (Néosynephrine, Europhta, France). The animals were then anesthetized by inhalation of isoflurane (Axience, France) and placed in front of the SD-OCT imaging device (Bioptigen 840 nm HHP; Bioptigen, North Carolina, USA). Eyes were kept moisturized with 0.9% NaCl throughout the procedure. Images were acquired with the following parameters: Rectangular scan/1000 A-scan per B-scan/100B-scan 1 frame or 4B-scans 16 frames. The images acquired were processed with Fiji software (available at http://fiji.sc/Fiji). Retinal layer thickness was measured manually on this maximum projection image, along an axis perpendicular to the individual layers and 500 µm from the center of the optic nerve.

### Electroretinography

Mice were kept in the dark overnight and anesthetized with a mixture of ketamine (500 mg/kg) and xylazine (10 mg/kg). Their pupils were dilated with mydriaticum and the cornea was anesthetized by local application of oxybuprocaine. A small wire loop electrode contacting the cornea through a layer of lacrygel was used to record the retinal response, with needle electrodes placed in the cheeks and back used as the reference and ground electrodes, respectively. Body temperature was maintained at ~37 °C with a heating pad. The light stimulus was provided by a 150 W xenon lamp in a Ganzfeld stimulator (Multiliner Vision; Jaeger/Toennies, Hochberg, Germany). Dark-adapted responses were measured in darkness, during flash stimulation (0.1 and 0.2 cds/m^2^). Photopic cone ERGs were recorded in response to a flash (10 cds/m^2^) on a rod-suppressing background (25 cd)/m^2^) after five minutes of light adaptation. Responses were amplified and filtered (1 Hz-low and 300 Hz-high cutoff filters) with a one-channel DC-/AC-amplifier. Each dark-adapted or photopic ERG response is the mean of five responses from a set of five stimulatory flashes. Flicker ERGs were recorded at 10 and 20 Hertz.

### Histology and immunohistochemistry

Ush1 mutant mice and their wild-type littermates were perfused with 4% paraformaldehyde (PFA) in phosphate-buffered saline (PBS) under anesthesia with a mixture of ketamine (500 mg/kg) and xylazine (10 mg/kg). The eyes were oriented using a cauterizer prior to enucleation and fixed in 4% PFA in PBS overnight at 4 °C. They were then incubated successively in 10%, 20% and 30% sucrose/PBS for cryosections, or dissected in PBS for flat-mounted retinas. Eyes were embedded in Neg-50 medium for cryosectioning. Vertical retinal cryosections (12 µm) were layered on Superfrost glass slides. All the sections considered in this work were cut following superior-inferior axis in the optic nerve area. For immunolabeling, sections were rehydrated in PBS, and permeabilized with 0.5% Triton X-100, 0.25% Tween 20 and 2.5% fetal bovine serum in PBS. Processed sections were immunolabeled with antibodies against blue opsin (Merck-Millipore, 1:250); red/green opsin (Merck-Millipore, 1:250); Iba1 (Wako; 1:500); and secondary antibodies conjugated with Alexa Fluor 488 or Alexa Fluor 594 (Molecular Probes, 1:500). TUNEL staining was performed on full dorso-ventral cryosections with the QIA39 | FragEL™ DNA Fragmentation Detection Kit (Merck-Millipore). Cell nuclei were visualized by staining with 200 nM DAPI, and images were obtained with an Olympus Fluoview 1000 confocal microscope. For immunolabeling, flat-mount retinas were permeabilized in 1% Triton X-100, 0.5% Tween 20 and 5% bovine albumin serum in PBS. Flat-mounted retinas were immunolabeled with the polyclonal Iba1 antibody (Wako; 1:500) and a secondary antibody conjugated with Alexa Fluor 594. Cell nuclei were visualized by staining with 200 nM DAPI, and images were obtained with an Olympus Fluoview 1000 confocal microscope. Cone quantification was performed on entire dorso-ventral retinal cross section using PNA (Lectin PNA Alexa 594 conjugate Molecular Probes; 1:200) to count cone outer/inner segments. Microglial cells were quantified in flat-mounted retinas with IMARIS software.

### Statistics

Results have been statically analyzed performing Student’s t-test: (*), (**), and (***) denote *p* < 0.05, *p* < 0.01, and *p* < 0.005. Error bars are defined as the standard error of the mean. Imaging and TUNEL assays were done blinded as well as *in vivo* recording whenever possible.

## Electronic supplementary material

Supplementary Dataset 1

## References

[1] Mathur P, Yang J (2015). Usher syndrome: Hearing loss, retinal degeneration and associated abnormalities. Biochimica et biophysica acta.

[2] Petit C, Richardson GP (2009). Linking genes underlying deafness to hair-bundle development and function. Nature neuroscience.

[3] Richardson GP, de Monvel JB, Petit C (2011). How the genetics of deafness illuminates auditory physiology. Annual review of physiology.

[4] Lenassi E, Robson AG, Luxon LM, Bitner-Glindzicz M, Webster AR (2015). Clinical heterogeneity in a family with mutations in USH2A. JAMA Ophthalmol.

[5] Imtiaz F (2012). USH1G with unique retinal findings caused by a novel truncating mutation identified by genome-wide linkage analysis. Molecular vision.

[6] Ben Rebeh I (2010). Reinforcement of a minor alternative splicing event in MYO7A due to a missense mutation results in a mild form of retinopathy and deafness. Molecular vision.

[7] Astuto LM (2002). CDH23 mutation and phenotype heterogeneity: a profile of 107 diverse families with Usher syndrome and nonsyndromic deafness. American journal of human genetics.

[8] Zhou Q (2012). Evidence of genetic heterogeneity in Alberta Hutterites with Usher syndrome type I. Molecular vision.

[9] Mustapha M (2002). A novel locus for Usher syndrome type I, USH1G, maps to chromosome 17q24-25. Human genetics.

[10] Zein WM (2014). Cone responses in Usher syndrome types 1 and 2 by microvolt electroretinography. Investigative ophthalmology & visual science.

[11] Seeliger MW, Zrenner E, Apfelstedt-Sylla E, Jaissle GB (2001). Identification of Usher syndrome subtypes by ERG implicit time. Investigative ophthalmology & visual science.

[12] Ait-Ali N (2015). Rod-derived cone viability factor promotes cone survival by stimulating aerobic glycolysis. Cell.

[13] Yu DY (2004). Photoreceptor death, trophic factor expression, retinal oxygen status, and photoreceptor function in the P23H rat. Investigative ophthalmology & visual science.

[14] Geller AM, Sieving PA (1993). Assessment of foveal cone photoreceptors in Stargardt’s macular dystrophy using a small dot detection task. Vision research.

[15] Tian C (2010). Ush1c gene expression levels in the ear and eye suggest different roles for Ush1c in neurosensory organs in a new Ush1c knockout mouse. Brain research.

[16] Johnson KR (2003). Mouse models of USH1C and DFNB18: phenotypic and molecular analyses of two new spontaneous mutations of the Ush1c gene. Human molecular genetics.

[17] Williams DS (2009). Harmonin in the murine retina and the retinal phenotypes of Ush1c-mutant mice and human USH1C. Investigative ophthalmology & visual science.

[18] Liu X, Udovichenko IP, Brown SD, Steel KP, Williams DS (1999). Myosin VIIa participates in opsin transport through the photoreceptor cilium. J Neurosci.

[19] Libby RT, Steel KP (2001). *Electroretinograp*hic anomalies in mice with mutations in Myo7a, the gene involved in human Usher syndrome type 1B. Investigative ophthalmology & visual science.

[20] Colella P (2013). Myosin7a deficiency results in reduced retinal activity which is improved by gene therapy. PLoS One.

[21] Di Palma F (2001). Mutations in Cdh23, encoding a new type of cadherin, cause stereocilia disorganization in waltzer, the mouse model for Usher syndrome type 1D. Nature genetics.

[22] Libby RT, Kitamoto J, Holme RH, Williams DS, Steel KP (2003). Cdh23 mutations in the mouse are associated with retinal dysfunction but not retinal degeneration. Experimental Eye Research.

[23] Ball SL, Bardenstein D, Alagramam KN (2003). Assessment of retinal structure and function in Ames waltzer mice. Investigative ophthalmology & visual science.

[24] Haywood-Watson RJ (2006). Ames Waltzer deaf mice have reduced electroretinogram amplitudes and complex alternative splicing of Pcdh15 transcripts. Investigative ophthalmology & visual science.

[25] Kikkawa Y (2003). Mutations in a new scaffold protein Sans cause deafness in Jackson shaker mice. Human molecular genetics.

[26] Volland S (2015). Three-dimensional organization of nascent rod outer segment disk membranes. Proceedings of the National Academy of Sciences of the United States of America.

[27] Sahly I (2012). Localization of Usher 1 proteins to the photoreceptor calyceal processes, which are absent from mice. The Journal of cell biology.

[28] Lentz JJ (2010). Deafness and retinal degeneration in a novel USH1C knock-in mouse model. Dev Neurobiol.

[29] Liu X, Ondek B, Williams DS (1998). Mutant myosin VIIa causes defective melanosome distribution in the RPE of shaker-1 mice. Nature genetics.

[30] Gibbs D (2004). Role of myosin VIIa and Rab27a in the motility and localization of RPE melanosomes. Journal of cell science.

[31] Gibbs D, Kitamoto J, Williams DS (2003). Abnormal phagocytosis by retinal pigmented epithelium that lacks myosin VIIa, the Usher syndrome 1B protein. Proceedings of the National Academy of Sciences of the United States of America.

[32] Lopes VS (2011). The Usher 1B protein, MYO7A, is required for normal localization and function of the visual retinoid cycle enzyme, RPE65. Human molecular genetics.

[33] Peng YW, Zallocchi M, Wang WM, Delimont D, Cosgrove D (2011). Moderate light-induced degeneration of rod photoreceptors with delayed transducin translocation in shaker1 mice. Investigative ophthalmology & visual science.

[34] Lefevre G (2008). A core cochlear phenotype in USH1 mouse mutants implicates fibrous links of the hair bundle in its cohesion, orientation and differential growth. Development (Cambridge, England).

[35] Caberlotto E (2011). Usher type 1G protein sans is a critical component of the tip-link complex, a structure controlling actin polymerization in stereocilia. Proceedings of the National Academy of Sciences of the United States of America.

[36] Lyubarsky AL, Daniele LL, Pugh EN (2004). From candelas to photoisomerizations in the mouse eye by rhodopsin bleaching *in situ* and the light-rearing dependence of the major components of the mouse ERG. Vision research.

[37] Froger N (2014). Taurine: The comeback of a neutraceutical in the prevention of retinal degenerations. Progress in retinal and eye research.

[38] Gaucher D (2012). Taurine deficiency damages retinal neurones: cone photoreceptors and retinal ganglion cells. Amino acids.

[39] Jammoul F (2009). Taurine deficiency is a cause of vigabatrin-induced retinal phototoxicity. Annals of neurology.

[40] Rapp LM, Thum LA, Anderson RE (1988). Synergism between environmental lighting and taurine depletion in causing photoreceptor cell degeneration. Experimental eye research.

[41] Weil D (1995). Defective myosin VIIA gene responsible for Usher syndrome type 1B. Nature.

[42] Schietroma C (2017). Usher syndrome type 1-associated cadherins shape the photoreceptor outer segment. The Journal of cell biology.

[43] Berg DJ (1996). Enterocolitis and colon cancer in interleukin-10-deficient mice are associated with aberrant cytokine production and CD4(+) TH1-like responses. J Clin Invest.

[44] Bonnet C, El-Amraoui A (2012). Usher syndrome (sensorineural deafness and retinitis pigmentosa): pathogenesis, molecular diagnosis and therapeutic approaches. Curr Opin Neurol.

[45] Alagramam KN (2001). Mutations in the novel protocadherin PCDH15 cause Usher syndrome type 1F. Human molecular genetics.

[46] Chrysostomou V, Valter K, Stone J (2009). Cone-rod dependence in the rat retina: variation with the rate of rod damage. Investigative ophthalmology & visual science.

[47] Shen J (2005). Oxidative damage is a potential cause of cone cell death in retinitis pigmentosa. J Cell Physiol.

[48] Mrugacz M, Szuminski M, Sredzinska-Kita D, Bakunowicz-Lazarczyk A (2010). [Estimation of morphology and function of the eye in Usher’s syndrome]. Klin Oczna.

[49] Schwartz SB (2005). Disease expression in Usher syndrome caused by VLGR1 gene mutation (USH2C) and comparison with USH2A phenotype. Investigative ophthalmology & visual science.

[50] Malm E, Ponjavic V, Moller C, Kimberling WJ, Andreasson S (2011). Phenotypes in defined genotypes including siblings with Usher syndrome. Ophthalmic Genet.

[51] Domanico D, Fragiotta S, Trabucco P, Nebbioso M, Vingolo EM (2012). Genetic analysis for two italian siblings with usher syndrome and schizophrenia. Case Rep Ophthalmol Med.

[52] Bernal S (2005). Clinical and genetic studies in Spanish patients with Usher syndrome type II: description of new mutations and evidence for a lack of genotype–phenotype correlation. Clin Genet.

[53] Aleman TS (2001). Macular pigment and lutein supplementation in retinitis pigmentosa and Usher syndrome. Investigative ophthalmology & visual science.

