# Supplementary material for: Cone degeneration is triggered by the absence of USH1 proteins but prevented by antioxidant treatments
Source: Sci Rep. 2018 Jan 31;8:1968. doi: 10.1038/s41598-018-20171-0 (PMC5792440; doi:10.1038/s41598-018-20171-0)
Supplement: Supplementary file 1 — Supplementary Dataset 1 [file 41598_2018_20171_MOESM1_ESM.doc]

# Title: Cone degeneration is triggered by the absence of USH1 proteins but is prevented by antioxidant treatments

**Authors:** Alix Trouillet^1^, Elisabeth Dubus^1^, Julie Dégardin^1^, Amrit Estivalet^1^, Ivana Ivkovic^1^, David Godefroy^1^, Diego García-Ayuso^2^, Manuel Simonutti^1^, Iman Sahly^3,4^, José A. Sahel^1,5,6,7^, Aziz El-Amraoui^3,4^, Christine Petit^3,4,8^, and Serge Picaud^1*^

SUPPLEMENTAL INORMATION

# Supplemental Figure S1: No drastic retinal thinning in albino *Ush1g^-/-^* BALB/cJ mice.

(a) OCT scans at 9-month-old reveal no major difference in retinal layers thickness of BALB/cJ *Ush1g*^-/-^ mice compared to control mice (a). Outer nuclear layer (b) and total retinal (c) thicknesses were not statistically different between control and mutant mice as expected in a context of cone degeneration, but they showed decrease tendencies in these aged *Ush1g*^-/-^ BALB/cJ mice, (b, c).


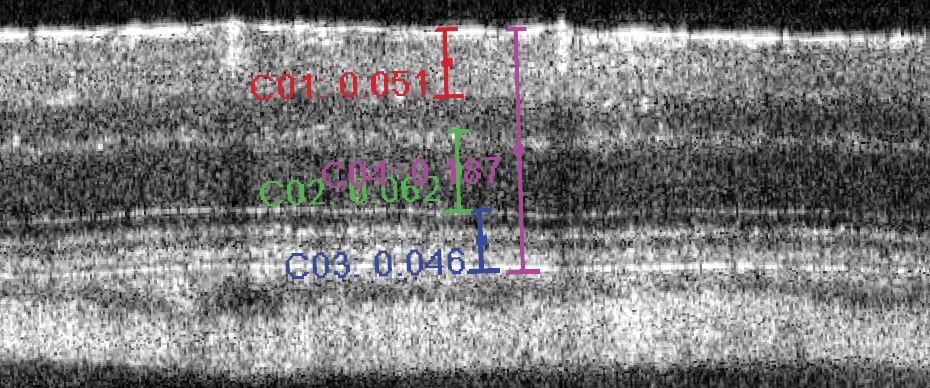

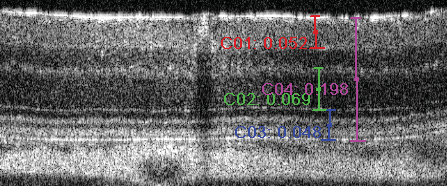
OCT scan in a wild-type BALB/cJ mouse OCT scan in an Ush1g−/− BALB/cJ mouse


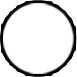


**a**

**0.070**


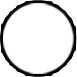


**b**

**Ventral**

**0.07**

**Dorsal**


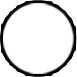


**c**

**0.20**

**Total retina thickness ( m)**

**Ventral Dorsal**

**0.20**

**0.065**

**Outer Nuclear Layer ( m)**

**0.06**

**0.19**

**0.19**

**0.060**

**0.05**

**0.18**

**0.18**

**0.055**

**control *Ush1g -/-***


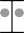


**0.04**

**control *Ush1g ^-/-^***

**0.17**

**control *Ush1g -/-***

**0.17**

**control *Ush1g -/-***

# Supplemental Figure S2: Cone dysfunction in albino *Ush1c*^-/-^ and *Ush1g^-/-^* BALB/cJ mice. (a,

1.
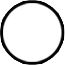
Quantification of scotopic a-wave and b-wave ERG amplitudes (a, c, 0.1 cds/m2) and flicker amplitudes (b, d, 20Hz) in three-month-old (mo3) and nine-month-old (mo 9) *Ush1g^-/-^* mice (a, b, red) or *Ush1c^-/-^* mice (c, d, blue) with their respective control BALB/cJ mice (grey), showing significantly lower amplitudes in the mutant mice on flickers 20Hz (*Ush1g^-/-^* mice mo 3: *p* = 0.0155 n=8; mo 9: *p* = 0.0079, n= 5 (*Ush1c^-/-^* mice mo 9: *p* = 0.0033, *n* = 10). In *Ush1c^-/-^* mice, an a-wave difference is also observed at mo 9 and a b-wave difference at mo 3 (*Ush1c^-/-^* mice a-wave mo 9: *p* =0.0451, n=5, b-wave mo 3: p= 0.0190, *n* = 10). The data are means ± SEM. (*), (**), and (***) denote *p* < 0.05, *p* < 0.01, and *p* < 0.005, respectively (Student’s *t*-test).


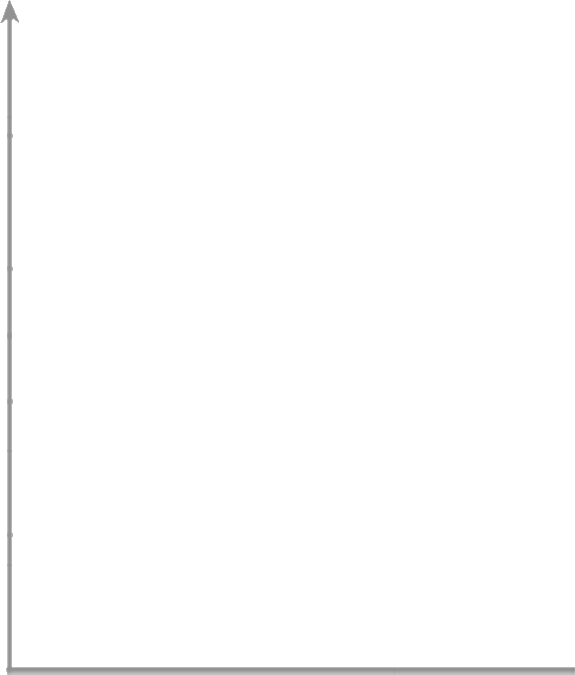

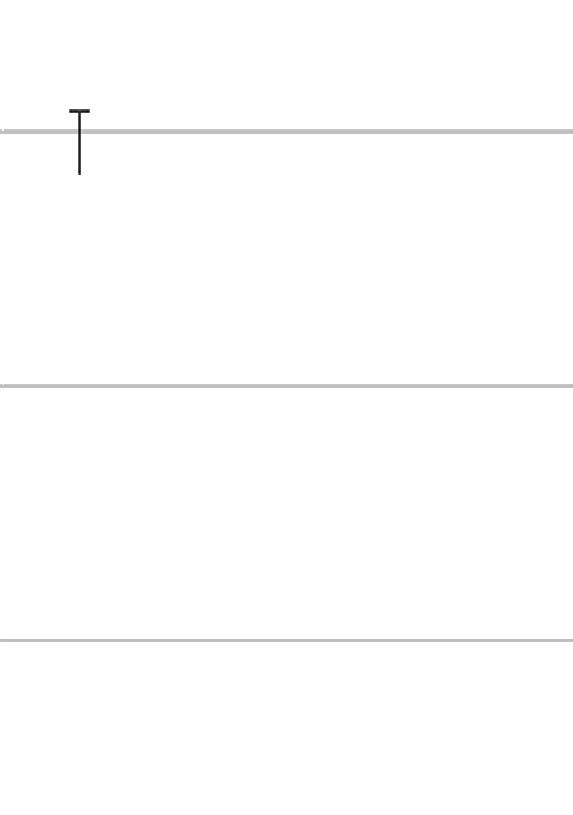

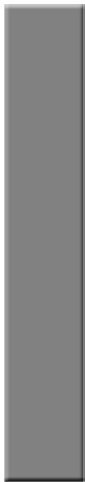

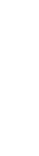

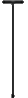

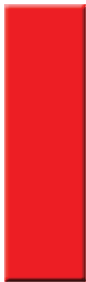

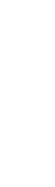

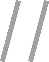

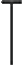

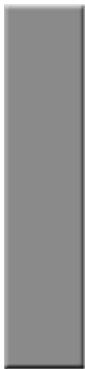

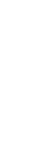

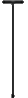

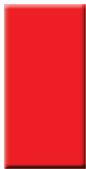


*

**

mo 3

mo 9

- 1.
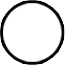
a-wave (V)


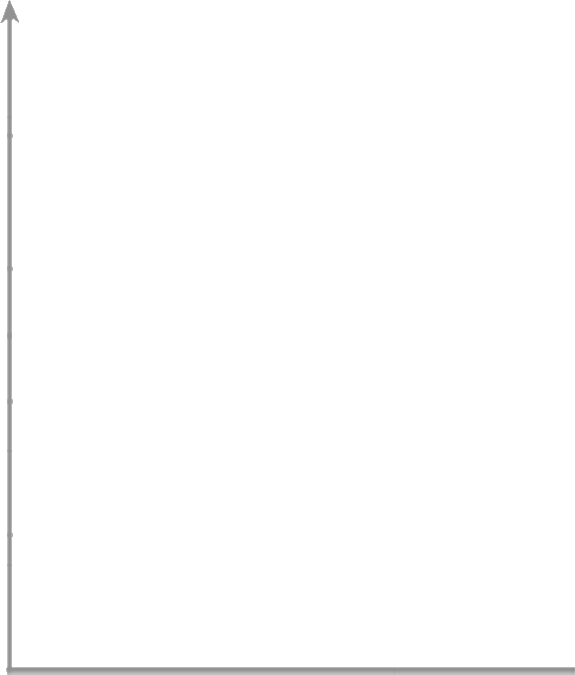

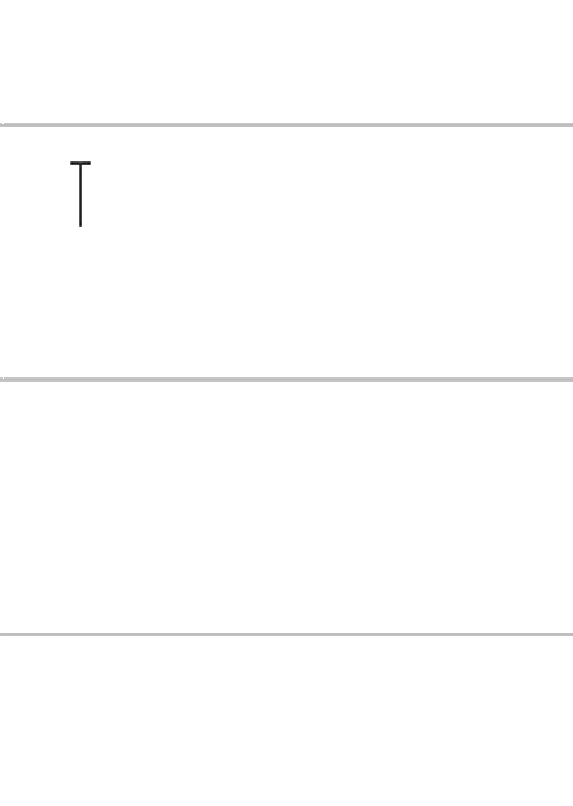

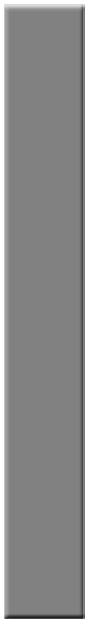

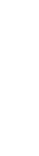

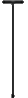

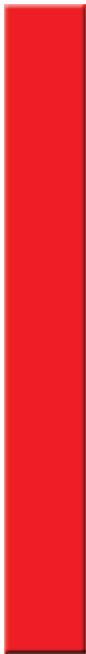

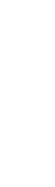

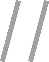

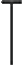

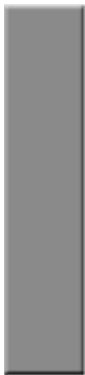

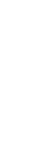

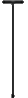

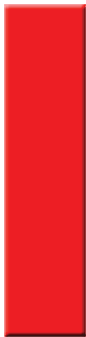

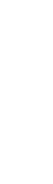


mo 3

mo 9

800 b-wave (V)

- 1. flickers 20 Hz (V)

400 600 20

200

400

10

200

0 0 0


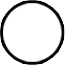
mo 3 mo 9


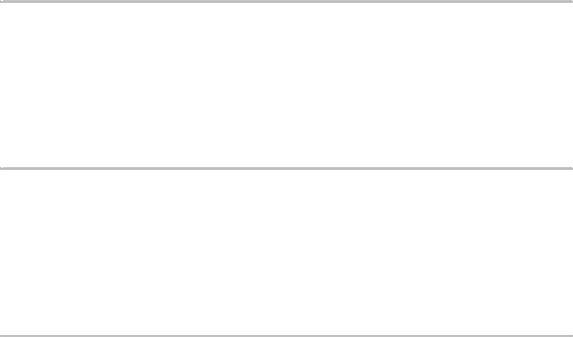

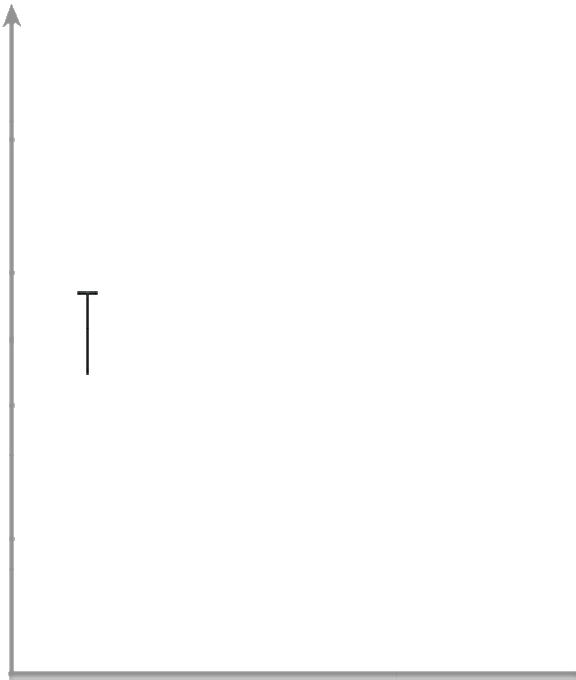

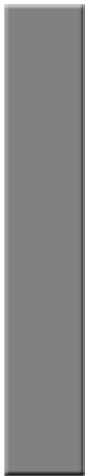

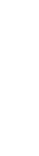

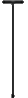

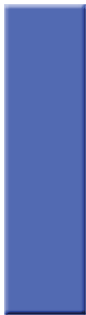

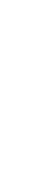

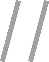

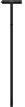

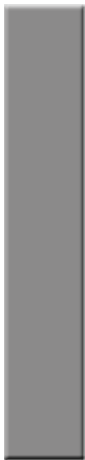

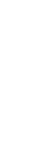

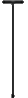

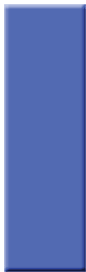


**


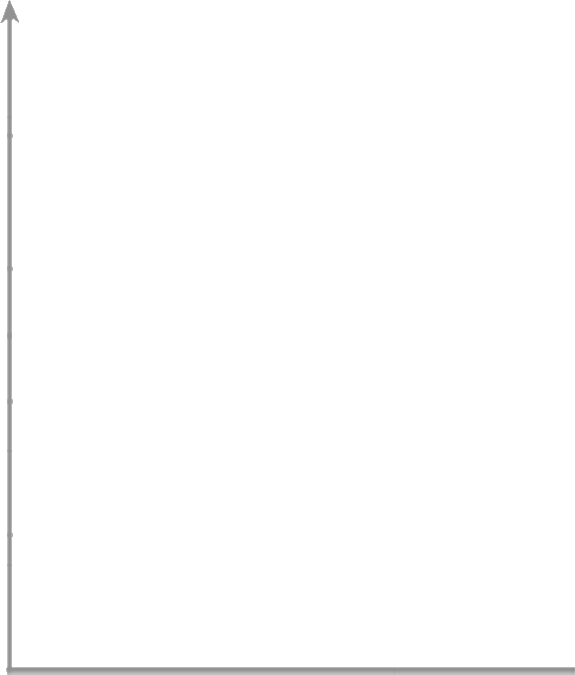

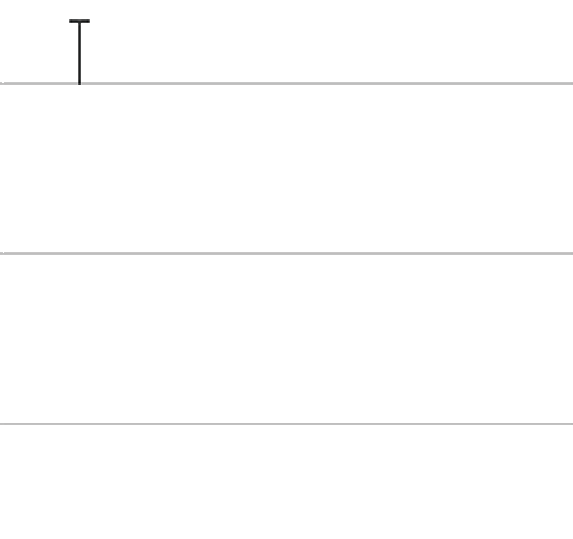

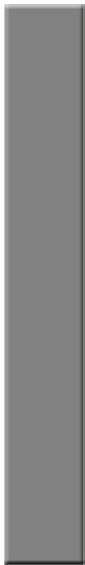

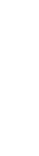

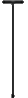

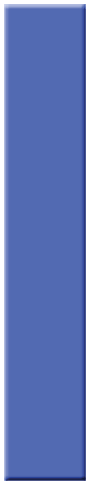

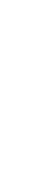

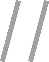

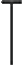

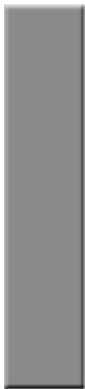

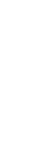

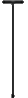

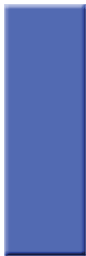

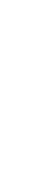


*

1.
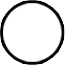
400 a-wave (V)


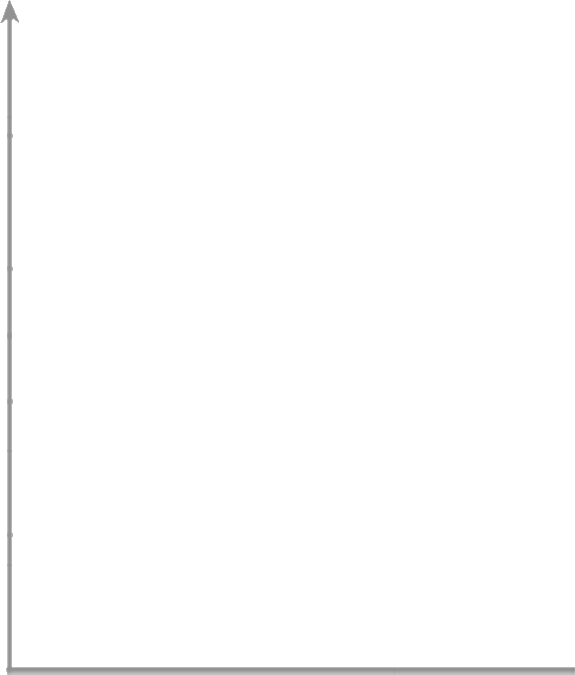

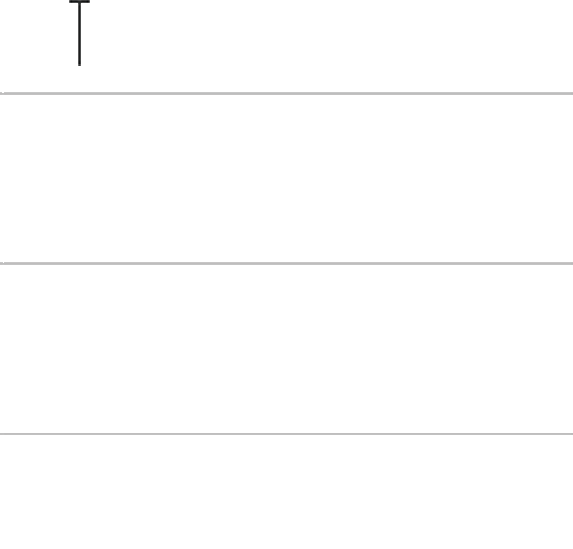

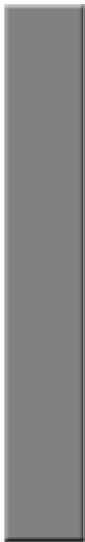

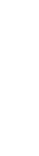

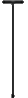

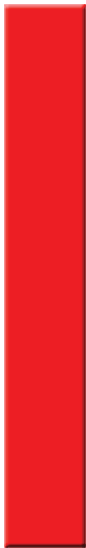

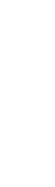

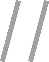

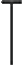

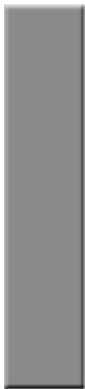

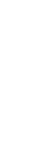

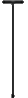

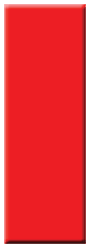

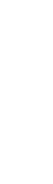

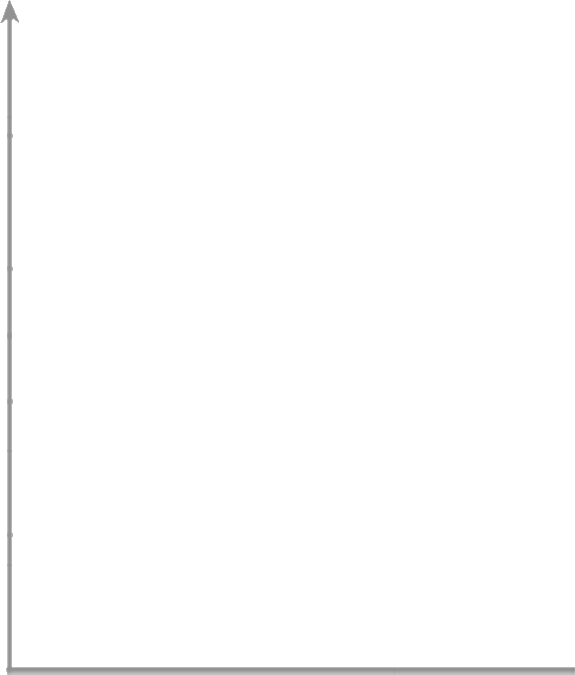

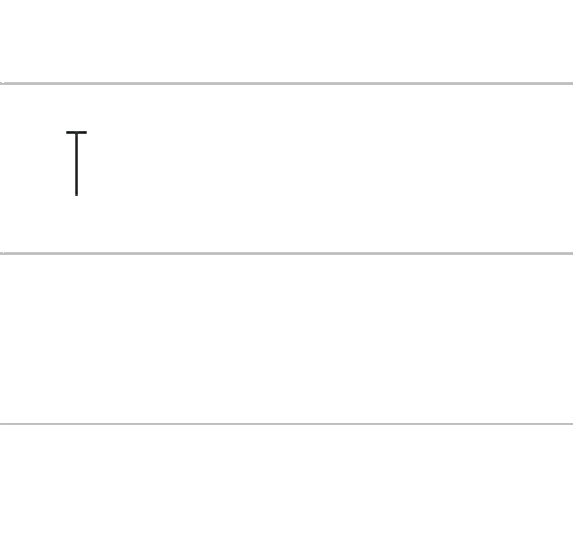

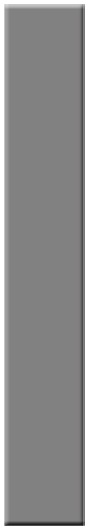

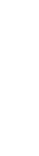

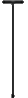

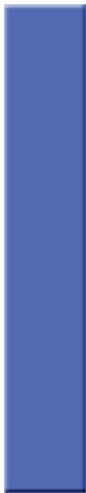

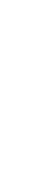

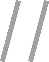

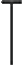

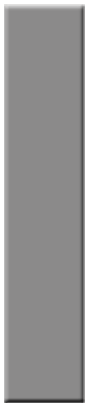

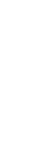

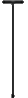

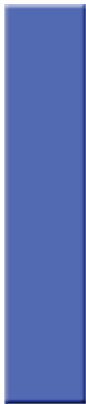

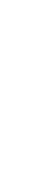


*

800 b-wave (V)

1. flickers 20 Hz (V)

300 600 20

200 400

10

100 200

0

mo 3 mo 9

0

mo 3

mo 9

0

mo 3 mo 9
